# Identification of immune-related genes that predict prognosis and risk of bladder cancer: bioinformatics analysis of TCGA database

**DOI:** 10.18632/aging.203333

**Published:** 2021-07-30

**Authors:** Liqiang Guo, Qiong Wu, Zhen Ma, Mingzhen Yuan, Shengtian Zhao

**Affiliations:** 1 Shandong Provincial Hospital Affiliated to Shandong First Medical University, Jinan, China; 2 Department of Gastroenterology, The Second Hospital of Shandong University, Jinan, China; 3 Cheeloo College of Medicine, Shandong University, Jinan, China; 4 Department of Urology, Shandong Provincial Hospital Affiliated to Shandong First Medical University, Jinan, China; 5 Binzhou Medical University, Binzhou, China

**Keywords:** immune-related genes, prognosis, bladder cancer, bioinformatics analysis, The Cancer Genome Atlas

## Abstract

Background: Bladder cancer (BLCA) is the major tumor of the urinary system, and immune-related genes (IRGs) contribute significantly to its initiation and prognosis.

Results: A total of 51 prognostic IRGs significantly associated with overall survival were identified. Functional enrichment analysis revealed that these genes were actively involved in tumor-related functions and pathways. Using multivariate Cox regression analysis, we detected 11 optimal IRGs (ADIPOQ, PPY, NAMPT, TAP1, AHNAK, OLR1, PDGFRA, IL34, MMP9, RAC3, and SH3BP2). We validated the prognostic value of this signature in two validation cohorts: GSE13507 (n = 165) and GSE32894 (n = 224). Furthermore, we performed a western blot and found that the expression of these IRGs matched their mRNA expression in TCGA. Moreover, correlations between risk score and immune-cell infiltration indicated that the prognostic signature reflected infiltration by several types of immune cells.

Conclusion: We identified and validated an 11-IRG-based risk signature that may be a reliable tool to evaluate the prognosis of BLCA patients and help to devise individualized immunotherapies.

Methods: Bioinformatics analysis was performed using TCGA and ImmPort databases. Cox regression was used to identify prognostic signatures. Two external GEO cohorts and western blotting of samples were performed to validate the IRG signature.

## INTRODUCTION

Bladder cancer (BLCA), with about 80,470 new patients in the United States in 2019, is the major malignant tumor of the urinary system [1]. Approximately 25% of patients are muscle-invasive or metastatic bladder cancer when they are initially diagnosed [2, 3]. Surgical resection is the main treatment for localized BLCA [4], whereas systematic chemotherapy is the preferred treatment for advanced and metastatic BLCA [5]. Despite these aggressive therapies, the five-year overall survival of bladder cancer remains less than 20% [6]. Thus, it is critical to explore alternative treatments and to determine promising prognostic indicators in BLCA.

At present, the molecular mechanism of BLCA has not yet been described, while growing evidence has revealed that immune-related genes (IRGs) and immune cell infiltration, play critical roles in the pathogenesis and progression of BLCA [7, 8]. In recent years, immune checkpoint therapies have provided a promising opportunity for the treatment of advanced BLCA patients [9]. Unfortunately, only approximately 20% of platinum-refractory and previously untreated patients may benefit from immunotherapy [10]. Therefore, it is imperative to screen and detect immunotherapy response and prognostic predictors to predict prognosis and risk of bladder cancer.

In this study, we used transcriptome data from TCGA to construct a prognostic signature of 11 differentially expressed IRGs. The prognostic IRG-based signature was further validated in two independent GEO datasets and in proteomics data from our samples. The underlying regulatory mechanisms of IRGs were explored using bioinformatics methods. This immunogenomic signature may be a reliable tool for individualized prediction of prognosis in BLCA patients.

## RESULTS

### Identification of differentially expressed IRGs

**Figure 1:**
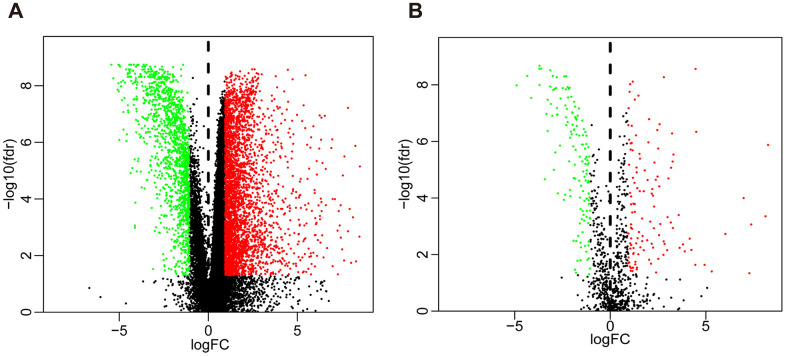
**Volcano plot of differentially expressed genes and immune-related genes.** (**A**) Volcano plot of differentially expressed genes between bladder cancer (BLCA) and non-tumor tissues. (**B**) Volcano plot of differentially expressed immune-related genes between bladder cancer (BLCA) and non-tumor tissues. The green dots represent downregulated genes, the red dots represent upregulated genes, and the black dots represent genes that were not significantly differentially expressed.

Compared with normal tissues, we identified 4876 differentially expressed genes (DEGs) in BLCA tissues including 3453 upregulated and 1423 downregulated genes (Figure 1A and Supplementary Figure 1A). We extracted 120 upregulated and 140 downregulated IRGs corresponding to those identified in the ImmPort database (Figure 1B and Supplementary Figure 1B). In addition, we performed GO functional enrichment and KEGG pathway analyses in 260 differentially expressed IRGs (DEIRGs). The top ten functional annotations were shown in Supplementary Table 1. The DEIRGs were mostly enriched in cell migration, leukocyte migration, extracellular matrix, receptor complex, receptor ligand activity and cytokine activity (Supplementary Figure 2A–2C). Furthermore, cytokine–cytokine receptor interactions were enriched in the KEGG pathways (Supplementary Figure 2D).

### Identification of prognosis-associated DEIRGs

**Figure 2:**
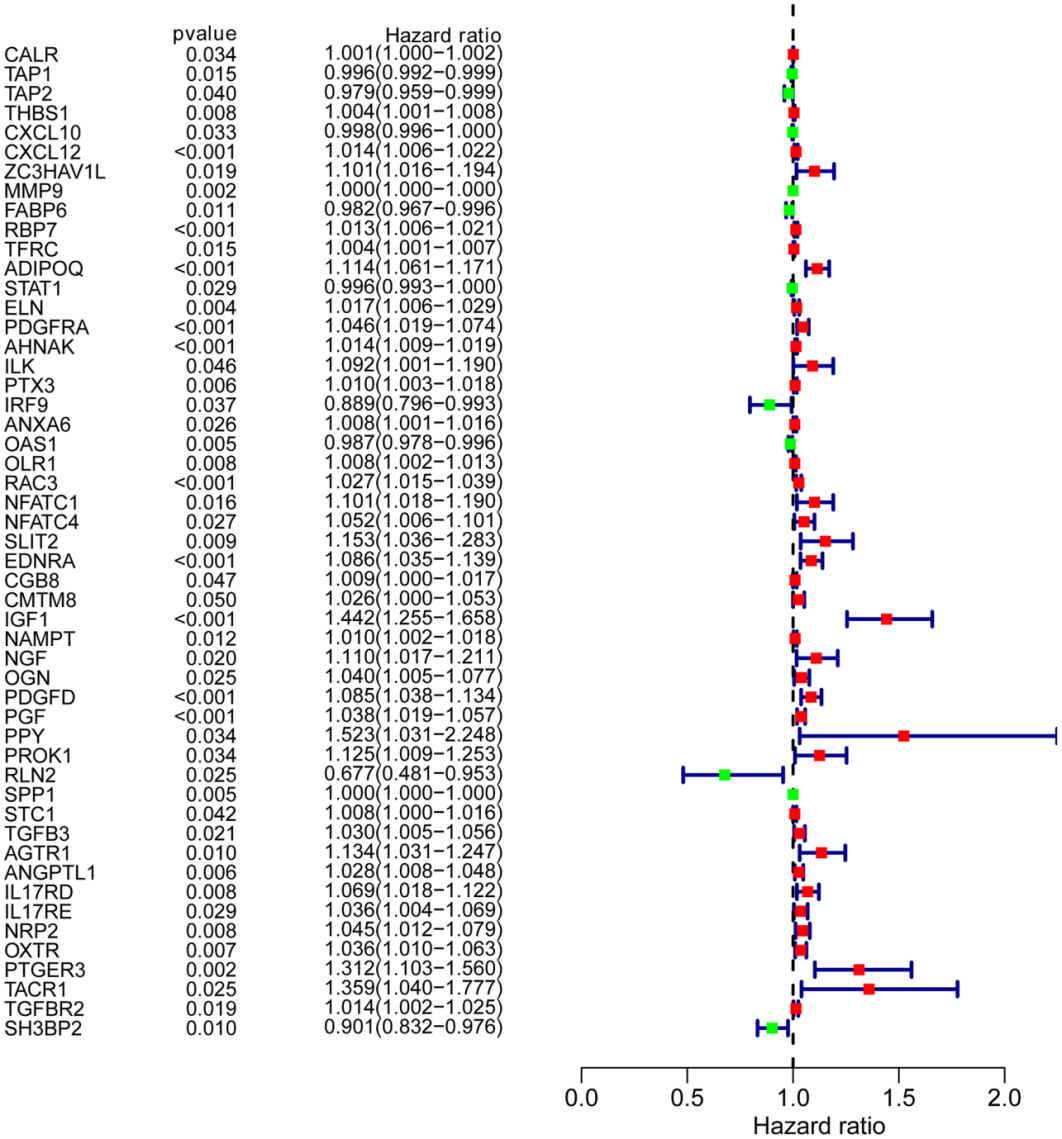
**Prognosis-associated DEIRGs.** Forest plot of hazard ratios showing the prognostic values of IRGs.

**Figure 3:**
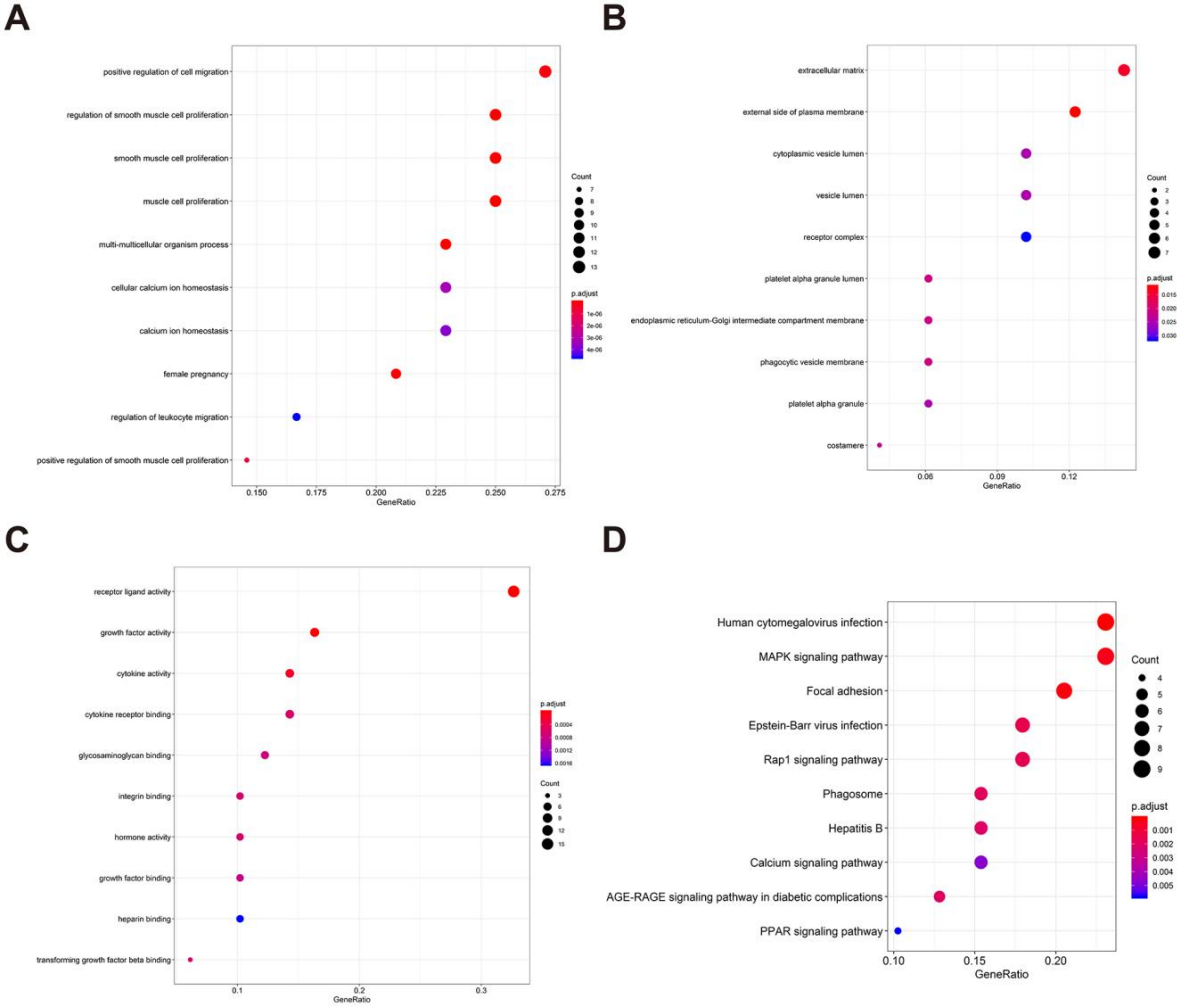
**Gene functional enrichment of prognosis-associated DEIRGs.** (**A**) The top ten most significant biological processes in the gene ontology. (**B**) The top ten most significant cellular components in the gene ontology. (**C**) The top ten most significant molecular functions in the gene ontology. (**D**) The top ten most significant KEGG pathways.

To determine possible prognosis-associated DEIRGs, univariate Cox regression analysis of DEIRGs were performed in the present study. After screening, we identified 51 DEIRGs that significantly correlated with the overall survival of BLCA patients (Figure 2). Similar to the results for the DEIRGs, we found that these prognosis-associated DEIRGs were most enriched in cell migration, cell proliferation, extracellular matrix, receptor ligand activity and growth factor activity (Supplementary Table 2 and Figure 3A–3C). Human cytomegalovirus infection and the MAPK signaling pathway were most enriched in the KEGG analysis (Figure 3D).

### Significant modular analysis based on a PPI network

**Figure 4:**
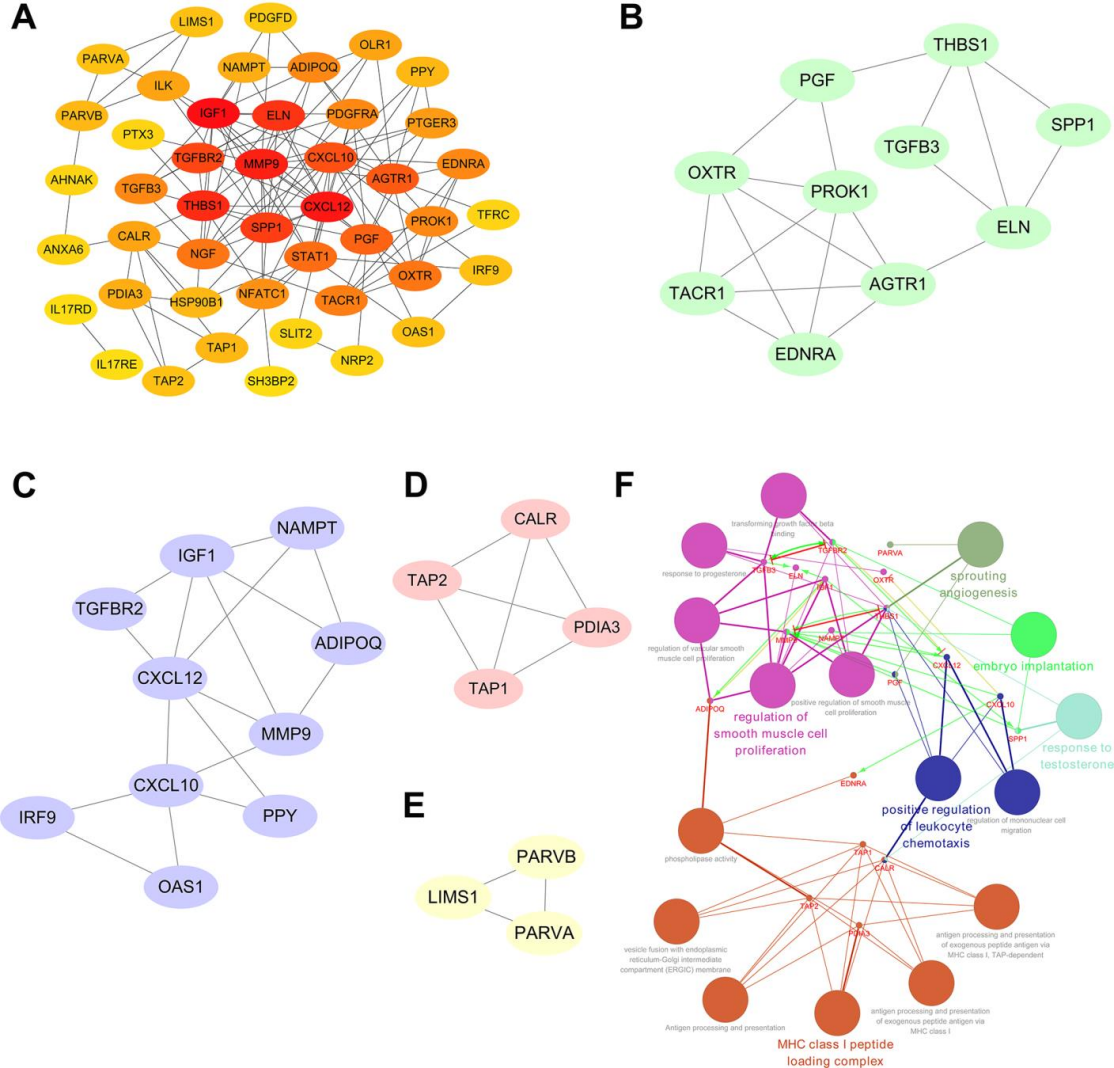
**Significant modular analysis and functional enrichment analysis based on the PPI network.** (**A**) Construction of a PPI network using a total of 51 prognosis-associated DEIRGs. (**B**) The most significant module subgroup of the hub genes, identified by MCODE plug-in, contains ten genes. (**C**) Module 2 contains ten hub genes. (**D**) Module 3 contains four hub genes. (**E**) Module 4 contains three hub genes. (**F**) Functional enrichment analysis of the 27 hub genes in the PPI network.

A PPI network was established utilizing the 51 prognosis-associated DEIRGs, shown in Figure 4A. In the PPI network, 27 hub genes were identified by the MCODE plugin of Cytoscape. When the *k*-core = 2, four significant module subgroups were obtained and were showed in different colors, and the most important modules, including *THBS1, PGF, SPP1, TGFB3, ELN, OXTR, PROK1, AGTR1, TACR1,* and *EDNRA*, were marked in green (Figure 4B–4E). As shown in Figure 4F, functional annotation indicated the 27 hub genes were mostly related in sprouting angiogenesis, positive regulation of leukocyte chemotaxis, regulation of smooth muscle cell proliferation, MHC class I peptide loading complex, response to testosterone, and embryo implantation.

### Construction of a transcription factor (TF) regulatory network

**Figure 5:**
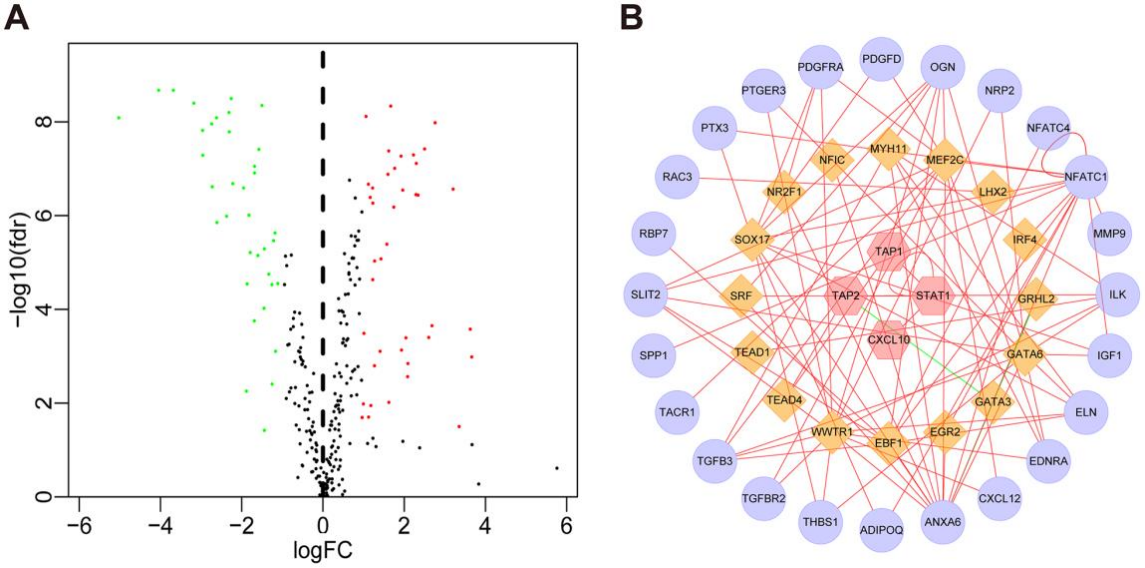
**TF regulatory network.** (**A**) Volcano plot of differentially expressed TFs. The green dots represent downregulated TFs, the red dots represent upregulated TFs, and the black dots represent TFs that were not significantly differentially expressed. (**B**) Regulatory network of TFs and IRGs; the yellow nodes represent TFs that correlated with the IRGs, the red nodes represent IRGs with hazard ratios < 1 (*p* < 0.05), the purple nodes represent IRGs with hazard ratios > 1 (*p* < 0.05) (correlation coefficient > 0.4 and *p* < 0.05), the green lines indicate negative regulatory relationships, and the red lines indicate positive regulatory relationships.

Next, we investigated the relationships between the DEIRGs in BLCA and the cancer-associated transcription factors (TFs). In total, we identified 77 differentially expressed TFs between BLCA (n = 414) and para-cancer tissues (n = 19) (Figure 5A, Supplementary Figure 3). Subsequently, using correlation scores > 0.4 and p values <0.05 as reference values, combined with 16 TFs and 51 DEIRGs, we constructed a TF regulatory network to illustrate the correlation between TFs and IRGs (Figure 5B).

### Construction of a prognostic risk model

**Figure 6:**
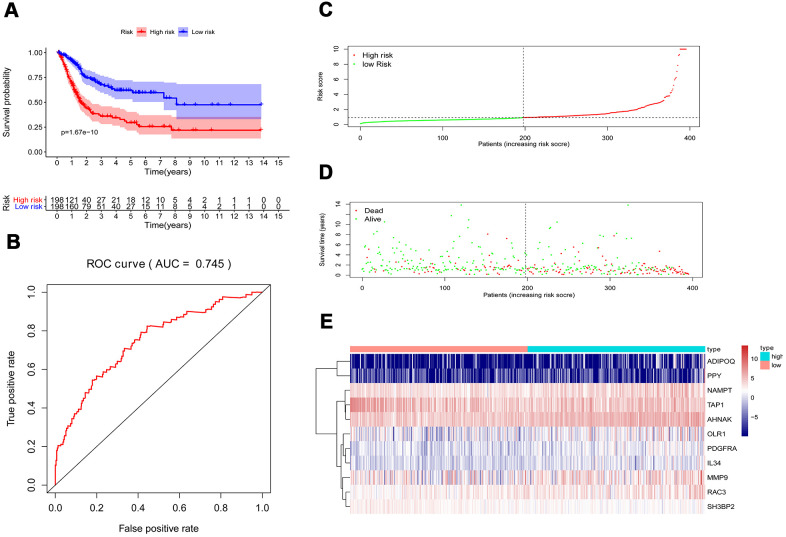
**Prognostic risk model of the cohort.** (**A**) Kaplan–Meier curve analysis showed that patients with a high-risk score were correlated with a worse survival outcome (*p* < 0.05). (**B**) ROC curve analysis of the prognostic risk model. (**C**) Risk score distribution of patients in the prognostic risk model in the cohort. (**D**) Survival status scatter plots for patients in the prognostic risk model. (**E**) Heatmap showing the distribution of the expression of the 11 immune-related genes in the cohort.

Multivariate Cox regression analysis were performed to calculate a risk score for each patient as follows: Risk score = (−0.0067 ×*TAP1*(expression)) + (0.0004 ×*MMP9*(expression)) + (0.0616 ×*ADIPOQ*(expression)) + (0.0359 ×*PDGFRA*(expression)) + (0.01261 ×*AHNAK*(expression)) + (0.0295 ×*RAC3*(expression)) + (0.0066 ×*OLR1*(expression)) + (0.0263 ×*IL34*(expression)) + (0.0149 ×*NAMPT*(expression)) + (0.0172 ×*PPY*(expression)) + (−0.0720 × *SH3BP2*(expression)). Based on the risk scores, patients with bladder cancer were divided into high-risk (n = 186) and low-risk (n = 185) groups. Kaplan–Meier analysis revealed that the survival rate significantly favored the low-risk group (*p* < 0.001) (Figure 6A). The AUC of the ROC curve was 0.745, suggesting moderate effectiveness for the prognostic risk model for monitoring survival (Figure 6B). Figure 6C–6E represented the risk scores, survival status, and heatmap of the ten IRGs between the two groups.

### Independent prognostic value of the risk model of the cohort

**Figure 7:**
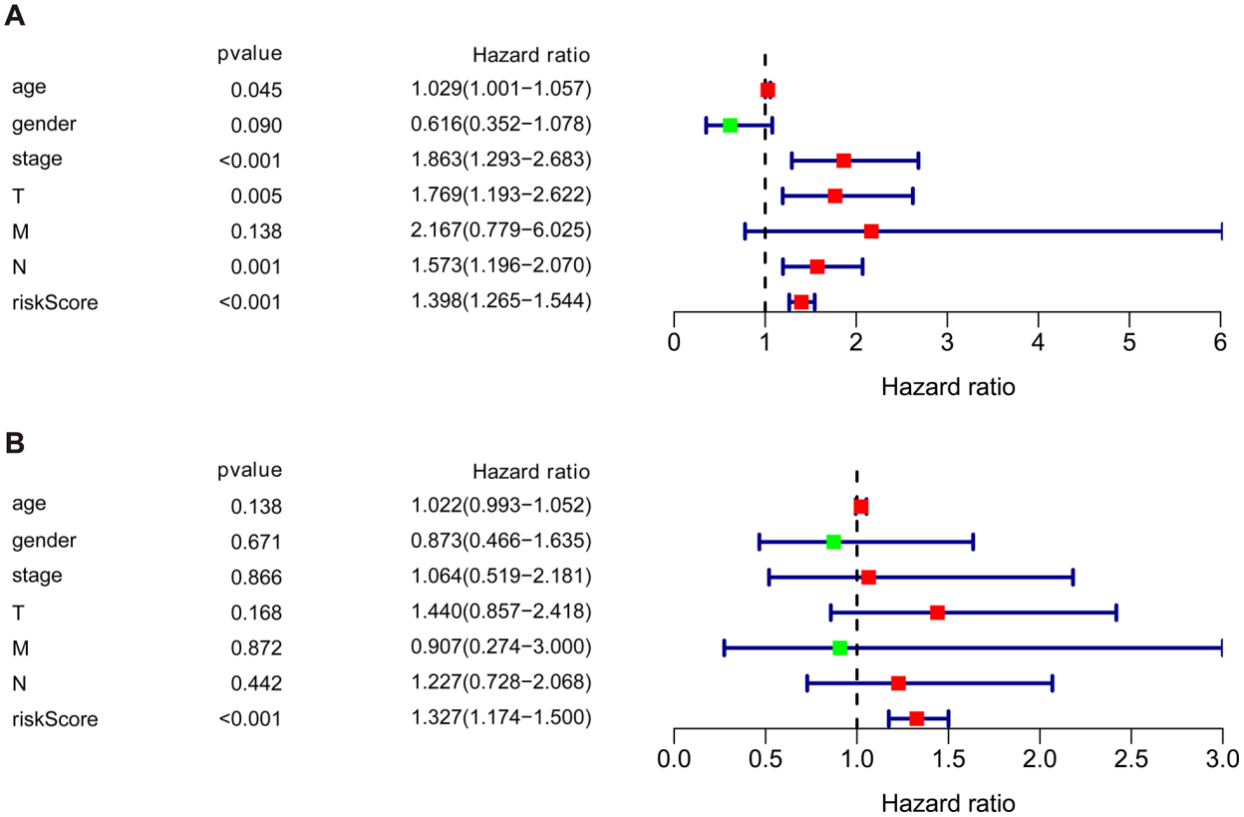
**Univariate and multivariate independent prognostic analysis of the cohort.** (**A**) Univariate Cox regression analysis showed clinical stage, tumor, lymph nodes, and risk score were associated with the prognosis of BLCA patients. (**B**) Multivariate Cox regression analysis revealed that the risk score was independently associated with OS in the cohort.

We then performed univariate and multivariate Cox regression analysis to determine the efficacy of risk score derived from our prognostic risk model as an independent predictor after adjusting for other clinical parameters (Table 1). Univariate analysis revealed that age, clinical stage, tumor stage (T), lymph node (N), and risk score were significantly related to the prognosis of BLCA patients (*p* < 0.05). Multivariate Cox regression analysis demonstrated that the risk score was independently corelated with the overall survival in the cohort (*p* < 0.001) (Figure 7).

**Table 1:** Univariate and multivariate Cox regression analyses of the cohort.

	**Univariate analysis**			**Multivariate analysis**		
	**HR**	**95%CI**	**P-value**	**HR**	**95%CI**	**p-value**
Age	1.029	1.001-1.057	0.04545	1.022	0.993-1.052	0.13814
Gender	0.616	0.352-1.078	0.08953	0.873	0.466-1.635	0.67126
Stage	1.863	1.293-2.683	0.00084	1.064	0.519-2.181	0.86637
T	1.769	1.193-2.622	0.00453	1.440	0.857-2.418	0.16832
M	2.167	0.779-6.025	0.13822	0.907	0.274-3.000	0.87234
N	1.573	1.196-2.070	0.00121	1.227	0.728-2.068	0.44174
Risk score	1.398	1.265-1.544	4.33e-11	1.327	1.174-1.500	6.28e-06

HR, hazard ratio; CI, confidence interval; T, tumor stage; M, metastasis; N, Lymph nodes.

### External validation of the prognostic risk model in the GSE13507 and GSE32894 cohorts

**Figure 8:**
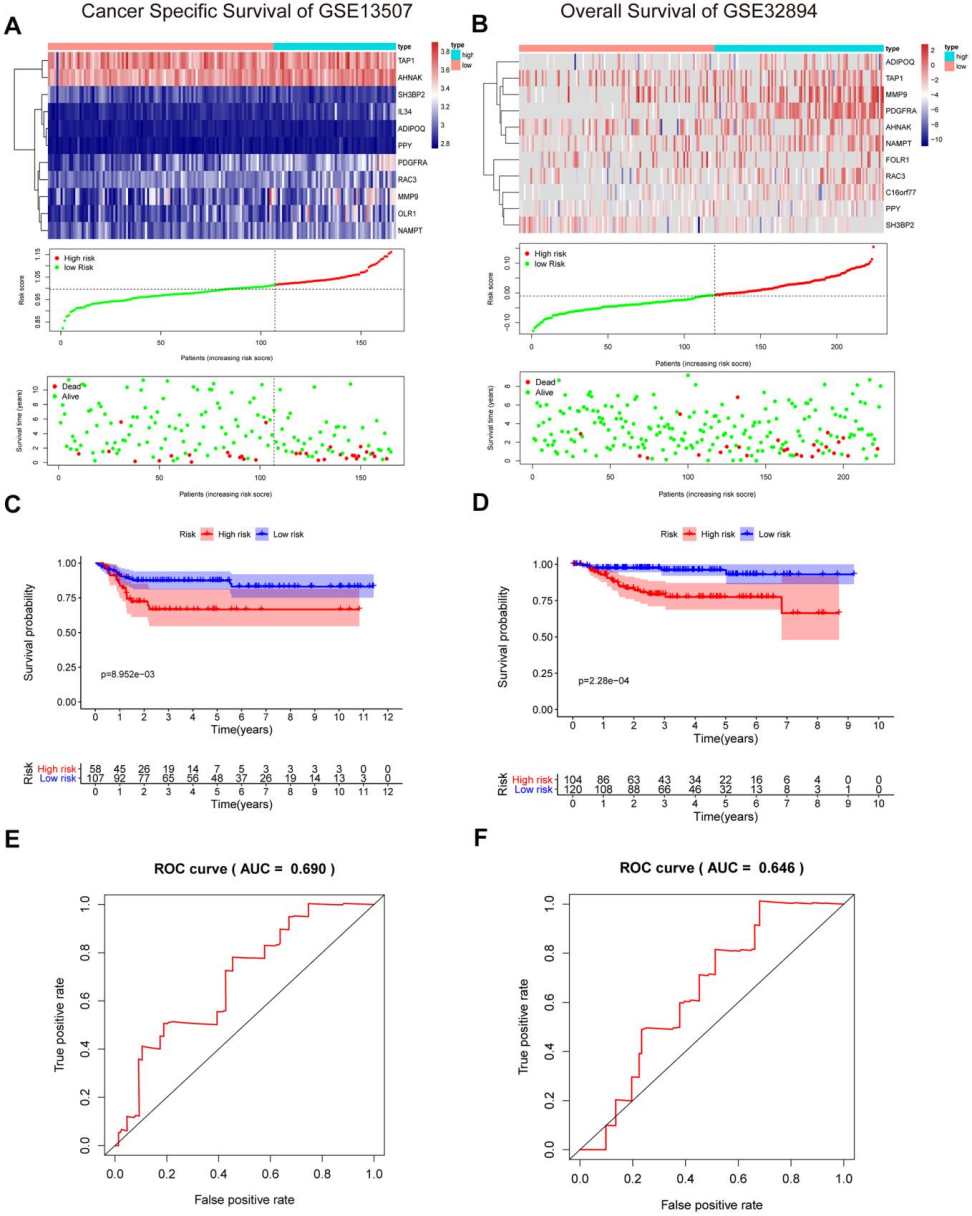
**Validation of the risk signature in GSE13507 and GSE32894 datasets.** (**A**, **B**) Heatmap of the 11 IRGs expression distribution, risk score distribution, and survival status between the low-risk group and high-risk group in the validation cohort. (**C**, **D**) Kaplan–Meier curve showed shorter survival time in the high-risk group patients. (**E**, **F**) ROC curve illustrated the prognostic value of the risk signature.

To validate the reliability of our IRG-based prognostic risk model, we utilized two external validation cohorts, GSE13507 and GSE32894. Similarly, patients in the high-risk group showed poorer survival than in the low-risk group (Figure 8A, 8B). In addition, the results were also shown with a KM curve and a ROC curve (Figure 8C–8F). The cumulative results imply that the IRG-based prognostic risk characteristics based on IRGs can be used as a reliable prognostic model.

### Validation of the IRGs in the proposed signature by western blot

**Figure 9:**
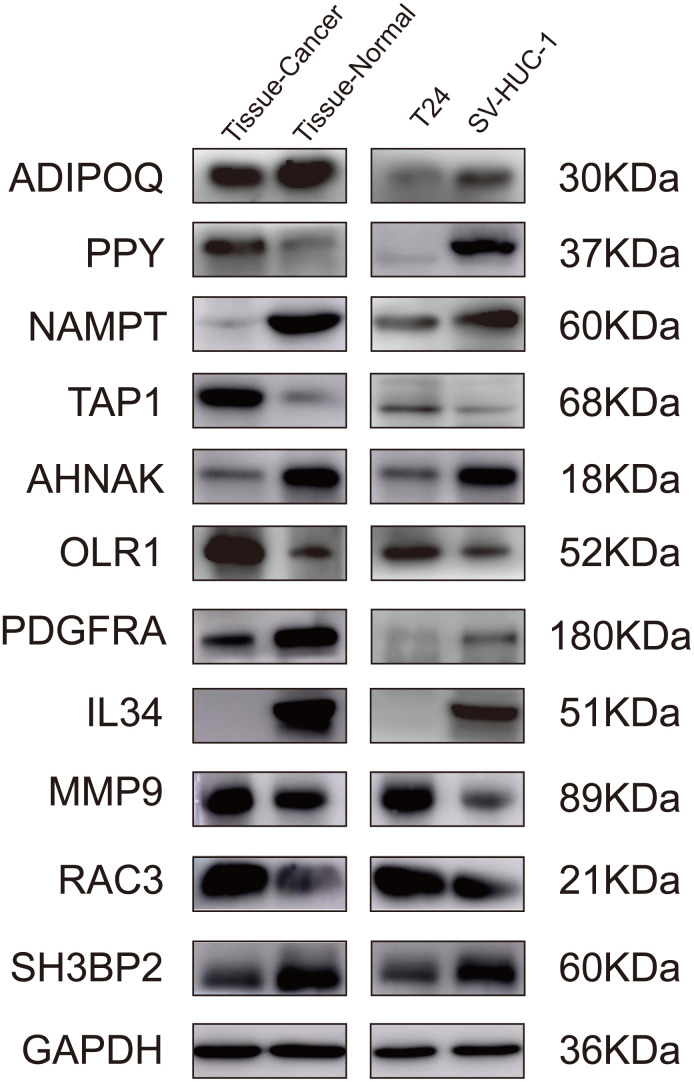
**Validation of the IRGs by western blot.** Validation of the IRGs by western blot on bladder cancer tissues and adjacent tissues, human normal bladder epithelial cells (SV-HUC-1), and a bladder cancer cell line (T24).

To further validate the proposed signature at the protein level, we performed western blotting on bladder cancer tissues, adjacent tissues, SV-HUC-1, and T24 cells lines. Comparing the gene levels of RNA-seq from TCGA dataset (data not shown) with the expression levels of WB protein, our results indicated that the protein levels of ADIPOQ, NAMPT, TAP1, OLR1, AHNAK, PDGFRA, IL34, MMP9 and RAC3 were consistent with their RNA expression trends (Figure 9).

### Clinical application of the prognostic model

**Figure 10:**
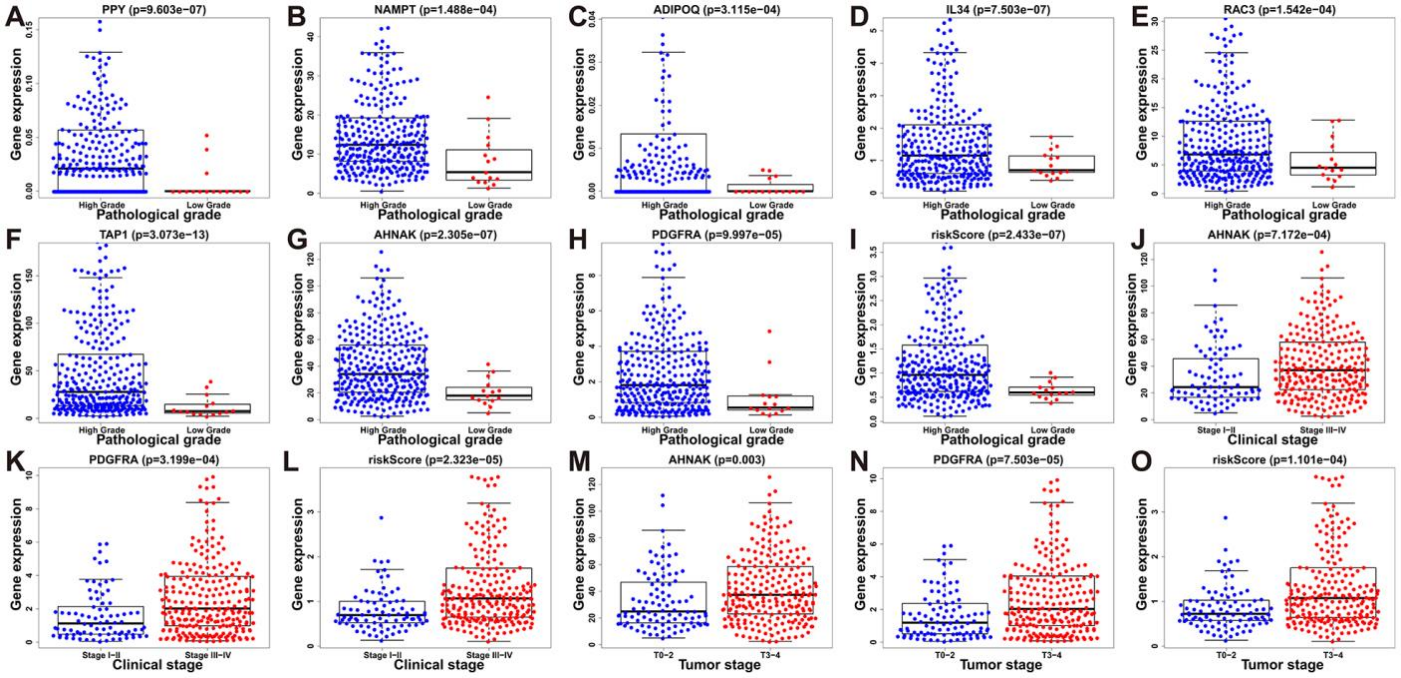
**Relationships between prognostic-model-associated IRGs and clinical variables in the TCGA cohort.** (**A**) *PPY* expression and pathological grade. (**B**) *NAMPT* expression and pathological grade. (**C**) *ADIPOQ* expression and pathological grade. (**D**) *IL34* expression and pathological grade. (**E**) *RAC3* expression and pathological grade. (**F**) *TAP1* expression and pathological grade. (**G**) *AHNAK* expression and pathological grade. (**H**) *PDGFRA* expression and pathological grade. (**I**) Risk score and pathological grade. (**J**) *AHNAK* expression and clinical stage. (**K**) *PDGFRA* expression and clinical stage; (**L**) Risk score and clinical stage. (**M**) *AHNAK* expression and tumor stage. (**N**) *PDGFRA* expression and tumor stage. (**O**) Risk score and tumor stage.

To assess the efficacy of our model in predicting the progression of BLCA, we evaluated the correlations between the risk signatures and clinical parameters including age, sex, pathological grade and clinical stage in the TCGA data cohort (Table 2). As the levels of PPY, NAMPT, ADIPOQ, IL34, RAC3, TAP1, AHNAK, PDGFRA and the risk score increased, the pathological grade of BLCA patients increased (p < 0.01) (Figure 10A–10I). With the increase of the other factors (risk score and PDGFRA and AHNAK levels), the clinical stage and the tumor stage of patients with BLCA also increased (all p < 0.01) (Figure 10J–10O). These results demonstrate that the dysregulation of the expression of the DEIRGs is significantly associated with the development of BLCA.

**Table 2:** Relationships between prognostic model associated IRGs and clinical variables of patients with BLCA.

**Variables**	**Age (≤65/≥65) t(p)**	**Gender (Female/Male) t(p)**	**Pathological grade (High grade/Low grade) t(p)**	**Clinical stage (3&4/1&2) t(p)**	**Tumor stage (T3&4/T1&2) t(p)**	**Lymph nodes (N1&2/N0) t(p)**
TAP1	1.132(0.259)	-0.104(0.917)	9.129(3.073e-13)	1.298(0.197)	1.006(0.316)	1.492(0.137)
MMP9	-0.974(0.331)	-0.888(0.376)	1.663(0.097)	-1.16(0.247)	-1.206(0.229)	-1.003(0.318)
ADIPOQ	-2.377(0.018)	1.167(0.246)	3.645(3.115e-04)	-0.339(0.735)	-0.662(0.509)	0.233(0.816)
PDGFRA	0.366(0.714)	1.446(0.150)	4.534(9.997e-05)	-3.645(3.199e-04)	-4.011(7.503e-05)	-1.492(0.137)
AHNAK	0.087(0.931)	0.901(0.369)	6.793(2.305e-07)	-3.435(7.172e-04)	-2.989(0.003)	-2.321(0.021)
OLR1	-0.016(0.987)	-0.369(0.712)	2.12(0.047)	-1.851(0.066)	-2.358(0.019)	-0.388(0.698)
RAC3	-1.8(0.073)	0.886(0.377)	4.227(1.542e-04)	-1.774(0.078)	-1.059(0.291)	-0.581(0.562)
IL34	-0.849(0.397)	0.352(0.725)	5.066(7.503e-07)	-1.974(0.049)	-2.444(0.015)	-2.053(0.042)
NAMPT	-0.698(0.486)	2.224(0.028)	4.5(1.488e-04)	-1.933(0.054)	-1.62(0.106)	0.682(0.495)
PPY	-1.385(0.167)	-0.174(0.862)	4.995(9.603e-07)	-1.524(0.128)	-1.795(0.074)	0.139(0.890)
SH3BP2	0.983(0.327)	-2.401(0.017)	-0.586(0.566)	2.384(0.019)	1.327(0.186)	1.974(0.049)
Risk Score	-1.426(0.155)	1.464(0.146)	5.272(2.433e-07)	-4.304(2.323e-05)	-3.926(1.101e-04)	-1.371(0.172)

*t*, t value determined using the Student *t* test; p, p value determined using the Student *t* test.

### Inferred immune cell fractions of the high- and low-risk groups

**Figure 11:**
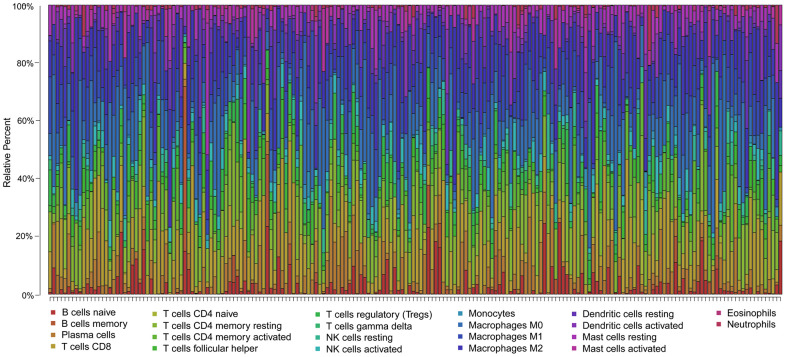
**Summary of the 22 immune cell subtypes estimated by the CIBERSORT algorithm.** The bar charts exhibit the cell proportions of BLCA patients and various colors represent the 22 immune cells with annotations below the legend.

**Figure 12:**
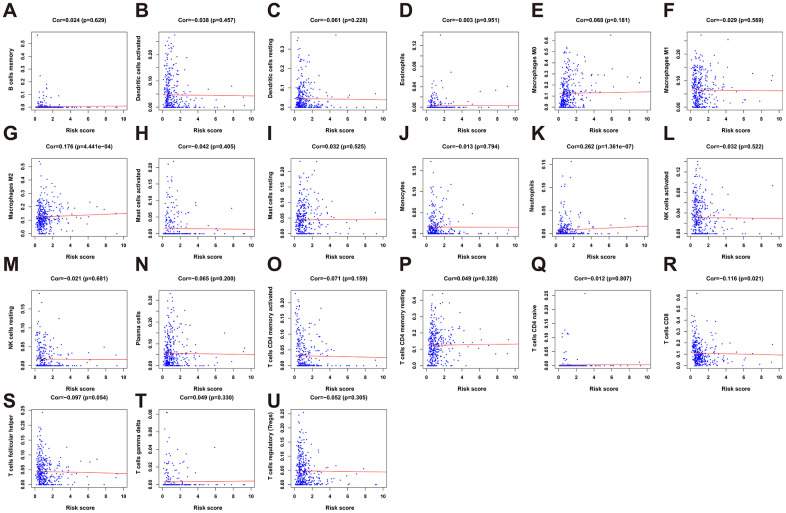
**The correlation between the risk score and immune cell infiltration in the cohort.** (**A**) B cells memory. (**B**) Dendritic cells activated. (**C**) Dendritic cells resting. (**D**) Eosinophils. (**E**) Macrophages M0. (**F**) Macrophages M1. (**G**) Macrophage M2. (**H**) Mast cell activated. (**I**) Mast cell resting. (**J**) Monocytes. (**K**) Neutrophils. (**L**) NK cells activated. (**M**) NK cells resting. (**N**) Plasma cells. (**O**) T cells CD4 memory activated. (**P**) T cells CD4 memory resting. (**Q**) T cells CD4 naïve. (**R**) T cells CD8. (**S**) T cells follicular helper. (**T**) T cells gamma delta. (**U**) T cells regulatory (Tregs).

To clarify whether our prognostic risk model can reflect the status of tumor immune microenvironment in BLCA patients, we evaluated the correlation between risk scores and immune cell infiltrations estimated by the CIBERSORT algorithm (Figure 11). The correlations between the risk score of the prognostic signature and immune cell infiltration are shown in Figure 12. As the risk score increased, the numbers of neutrophils, M2 macrophages, and CD8^+^ T cells in BLCA tissues increased (Figure 12).

## DISCUSSION

Although cancer immunotherapy has expanded the treatment possibilities for BLCA, only a subset of patients responds to immunotherapy [11–14]. Thus, it is crucial to identify immune-related biomarkers for the progression of BLCA patients to improve the effect of immunotherapy. Recent studies have reported genome-wide profiling investigating the role of multiple immune-related signatures in predicting tumor outcomes [15–17]. However, very few of these studies gained constructive therapeutic implications. Here, we established a robust immune-related risk signature of BLCA by integrated analysis of transcriptional profiles in TCGA. We also conducted external validation on overall survival rate and cancer-specific survival rate through two GEO datasets (GSE13507, n = 256; GSE32894, n = 308). Moreover, we performed western blotting in bladder tissues and cell lines to validate the IRGs at the protein level. As expected, the western blot demonstrated that the protein levels of the IRGs matched their RNA-seq levels derived from TCGA and GEO datasets.

Here we used univariate Cox regression analysis to assess the relationships between 260 DEIRGs and the prognosis of BLCA patients. We found that the expression of 51 DEIRGs significantly correlated with overall survival. To explore the underlying modulators related to these IRGs, a TF regulatory network comprising 77 differentially expressed TFs and DEIRGs that potentially regulate hub IRGs were constructed. Our results reveled that *STAT1*, *TAP1*, *TAP2* and *CXCL10* played central roles in this network, suggesting they are hub gene regulators. The transporter associated with antigen processing (*TAP*) is necessary for T-cell recognition [18]. Recent studies have demonstrated that *TAP* plays a critical role in cell differentiation, proliferation, the development and progression of cancer [19–21]. In addition, *TAP* detection has been recognized as a new independent indicator for the course of chemotherapy and clinical monitoring of several type of cancers [22, 23]. *STAT1* is considered as an oncogene that promotes cell adhesion, migration, and proliferation in bladder cancer [24]. Furthermore, the TF–IRG regulatory network emphasized that these hub TFs are closely associated with the prognostic signature genes, such as *ADIPOQ, PDGFRA, MMP9* and *RAC3*.

We then determined the risk-stratification value of these prognosis-related DEIRGs and identified 11 prognostic IRGs as potential prognostic indicators in clinical practice. Furthermore, our results revealed that the prognostic model was independent of clinical characteristics. Moreover, the signature illustrated the ability to discriminate the overall survival and cancer-specific survival in both the GSE13507 and GSE32894 datasets.

Our IRG-based signature highlighted eleven IRGs, *ADIPOQ, PPY, NAMPT, TAP1, AHNAK, OLR1, PDGFRA, IL34, MMP9, RAC3*, and *SH3BP2*. Several studies showed that upregulation of *MMP9* could promote the proliferation and invasion of bladder cancer [25, 26]. *ADIPOQ* has been proposed to be a mediator of obesity-associated metabolism and to have direct effects on the development and progression of various types of malignancies [27, 28]. Recent studies have identified *NAMPT* as a potential bladder cancer biomarker [29]. The subcellular localization of *AHNAK* exhibited different between BLCA tissues and normal tissues [30]. *LOX-1* was upregulated in 57% of bladder cancer cells, and was associated with tumor invasion and metastasis [31, 32]. Furthermore, expression of *PDGFRA* has been reported in BLCA specimens [33, 34]. Moreover, upregulation of *RAC3* in bladder cancer predicted an adverse clinical outcome and increased tumor immune response [35, 36].

Gene functional annotation indicated that our IRGs were involved in cell migration, cell proliferation, cytokine interactions, and chemokine pathways. Cytokines and chemokines, the gene products of transcription factors, played important roles in BLCA progression, and metastasis [37–39]. In addition, a PPI network was conducted to elucidate the regulatory mechanisms influence on the IRGs at the protein level. *MMP9, NAMPT, CXCL12, ADIPOQ, CXCL10, TAP1, TAP2,* and *STAT1* figured prominently in the PPI network. Functional annotation of the core genes derived from the PPI network also showed these hub genes were mainly enriched in sprouting angiogenesis, positive regulation of leukocyte chemotaxis, and regulation of smooth muscle cell proliferation.

Our results showed that after adjusting other clinical characteristics including age, clinical stage, T and M, the risk score could be used as an independent predictor. Multivariate analysis revealed that the risk score was independently associated with the overall survival in the cohort with a considerable hazard ratio. To determine the significance of the predictive values of the DEIRGs, we evaluated the correlations between the signatures and the clinicopathological factors age, sex, pathological grade, clinical stage, and tumor stage. We found that the levels of *PPY*, *NAMPT*, *ADIPOQ*, *IL34*, *TAP1*, *RAC3*, *PDGFRA* and *AHNAK*, combined with the risk scores, positively correlated with the progression of BLCA. Thus, combining this signature with other clinical factors may serve as a tool for predicting the prognosis of BLCA patients.

Accumulating evidence indicates that immune infiltration plays vital roles in the prognosis of BLCA patients [40, 41]. Immune infiltration is an important determinant of treatment response and prognosis in BLCA patients, which is further supported by the findings of our present statistical analyses showing that the risk score positively correlated with the infiltration of tumors with CD8+ T cells, M2 macrophages, and neutrophils. The results establish the reliability of our signature to predict the prognoses of patients with BLCA. Limitations of our study include the intrinsically limited information acquired from bioinformatics analysis of transcriptome data, which may not reflect the entirety of the pathologically significant aspects of the antitumor immune response. In addition, as a retrospective study, our results still have a bias because of their heterogeneity, and so further preclinical and clinical investigations are required to identify the specific mechanisms of the effects of IRGs on BLCA.

In conclusion, we identified and validated an 11-IRG-based risk signature. The IRGs were mainly involved in tumor-related functions and pathways. Our immunogenomic signature may be a reliable tool to evaluate the prognosis of BLCA patients and help guide individualized immunotherapies. Nonetheless, further experiments are required to verify our present findings.

## MATERIALS AND METHODS

### Clinical samples and data collection

Clinical and transcriptome RNA-seq data of 433 BLCA samples, including 414 BLCA patients and 19 matched normal samples, downloaded from the TCGA cohort. IRGs downloaded from the ImmPort portal database included 2498 IRGs [42]. ImmPort is a curated dataset to promote the reuse of immunological research data generated by intramural and extramural programs of the United States National Institutes of Health, and privately funded investigators [43].

### Analysis of DEGs

DEGs were identified via the effective and convenient limma R package. We analyzed differential gene expression of all transcriptional data and IRGs using the cut-off values as follows: FDR < 0.05 and log_2_ |FC| > 1. The pheatmap R software package was used to display the DEGs and DEIRGs. Subsequently, prognosis-associated DEIRGs were analyzed using univariate and multivariate Cox analysis.

### Functional annotations and PPI network

GO enrichment analysis and KEGG pathway analysis were conducted with the clusterProfiler package [44]. In addition, we used STRING database to predict the PPI network of the prognosis-associated DEIRGs and to evaluate the degree of interactions between proteins [39]. Cytoscape (version 3.5) was utilized to visualize the interactive network data [45]. Cytoscape plug-ins including MCODE, ClueGO, and CluePedia were used as previously described [46–48]. Specifically, MCODE was used to screen the most significant modules of hub genes from the PPI networks with selection criteria as follows: node score cut-off = 0.2, degree cut-off ≥ 2, and *k*-score = 2. The GO and KEGG pathway analyses of the selected hub genes were visualized utilizing ClueGO and CluePedia plug-ins.

### Construction of a regulatory network linking TFs and IRGs

Cistrome Cancer is a comprehensive resource for predicting targets of TFs (http://cistrome.org/CistromeCancer/). Target prediction is based on integration of correlations of expression levels among samples in each cancer included in TCGA, as well as the genomic TF binding patterns included in the ChIP-seq data. The Cistrome Cancer database contains a list of 318 TFs [49]. We analyzed differentially expressed TFs and constructed a regulatory network linking TFs and IRGs.

### Construction of a prognostic risk model

Gene-weighted values were calculated using the regression coefficients of the multivariate Cox regression model. The equation used in this analysis was as follows:

$$\text{Risk}\;\text{score}\;=\sum_{i=1}^{n}\text{Coe}\;(\text{gene}\;i)*\text{Exp}\;(\text{gene}\;i)$$

“Coe (gene *i*)” represents the regression coefficient of gene *i* estimated from the multivariate Cox analysis, and “Exp (gene *i*)” is the expression of gene *i*. The prognostic risk model was used to calculate the risk score of each patient. Furthermore, KM curve was performed using the “survival” and “survminer” R packages.

### External validation of the proposed signature in the GSE13507 and GSE32894 cohorts

We utilized the same risk score formula and cut-off value in two external validation cohorts, GSE13507 and GSE32894, to validate the IRG-based prognostic risk model. The prognostic model was presented in each dataset that contained the differentially expressed genes, a risk plot, and the distribution of risk score. Additionally, we evaluated the area under the ROC curve with the “survival ROC” package to assess the survival differences in the external datasets.

### Validation of IRGs in the risk model by western blot

Bladder cancer tissues and normal adjacent tissues were obtained from six patients admitted to Shandong Provincial Hospital Affiliated to Shandong First Medical University. T24 cells were cultured in RPMI 1640, and SV-HUC-1 cells were maintained in F-12K medium. The medium was refreshed every other day. Bladder tissues and urothelial cell lines were lysed with RIPA buffer. 25 μg of protein quantified by BCA kit in the samples were subjected to 6–10% SDS-PAGE and transferred to a polyvinylidene fluoride membrane. The membrane was blocked with 5% skim milk and incubated with primary antibodies at 4° C overnight (Supplementary Table 3). After hybridization of secondary antibodies, the protein expression level was detected by the chemiluminescence method (Amersham Imager 600, GE, USA).

### Inference of immune cell infiltration in BLCA tissues

Here we estimated immune infiltration between high and low risk score groups with the LM22 (22 types of immune cells) signature file via the CIBERSORT algorithm. After 1000 permutations of CIBERSORT, the distribution of 22 subtypes of immune cells in each patient was exhibited, along with the *p*-values, correlation coefficients, and root mean squared error (RMSE), which evaluates the accuracy of the results of each sample.

### Statistical analysis

Statistical analysis was conducted using R software, and *p* < 0.05 indicated a significant difference. To evaluate the accuracy of the prognostic risk model, the “survivalROC” package was used to calculate the AUC. The *t* test was used to evaluate continuous variables, and the χ2 test was used to compare categorical variables. Multivariate analysis was performed to determine the independent prognostic significance of the immune-related risk signature. The relationships between the risk score and the clinical characteristics were evaluated using the “beeswarm” package.

## Supplementary Material

Supplementary Figures

Supplementary Tables
