# Supplementary material for: Identification of immune-related genes that predict prognosis and risk of bladder cancer: bioinformatics analysis of TCGA database
Source: Aging (Albany NY). 2021 Jul 30;13(15):19352–74. doi: 10.18632/aging.203333 (PMC8386543; doi:10.18632/aging.203333)
Supplement: Supplementary Figures [file aging-13-203333-s001.pdf]

SUPPLEMENTARY FIGURES

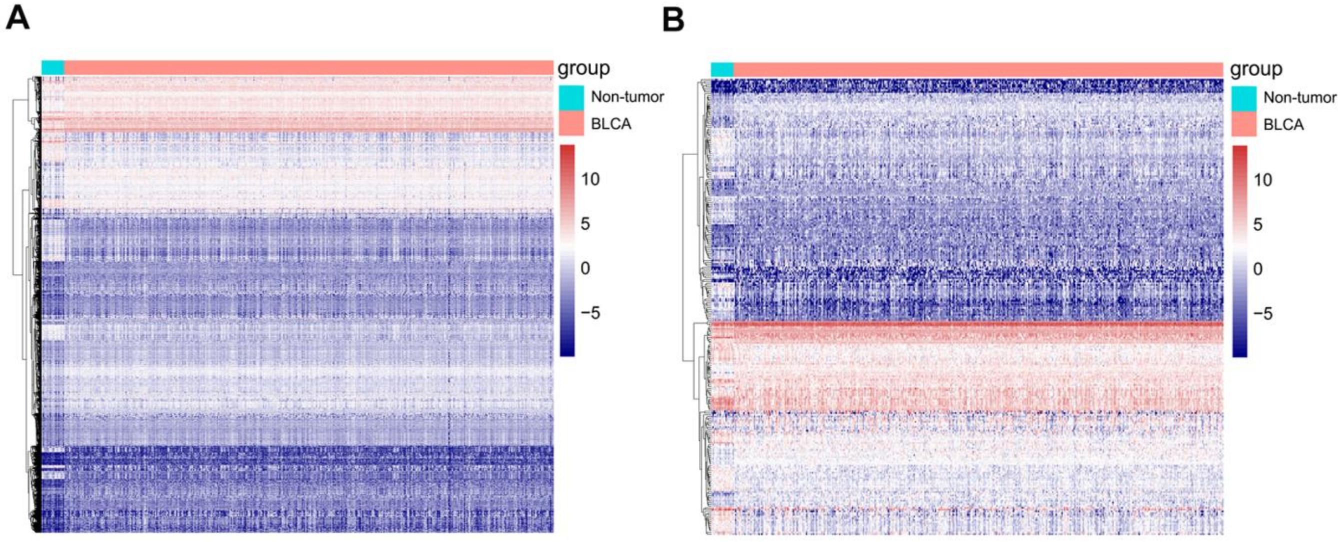

Supplementary Figure 1. Heatmap of differentially expressed genes and immune-related genes. (**A**) Heatmap of differentially expressed genes between bladder cancer (BLCA) and non-tumor tissues. (**B**) Heatmap of differentially expressed immune-related genes between bladder cancer (BLCA) and non-tumor tissues. The purple to red spectrum indicates low to high gene expression.

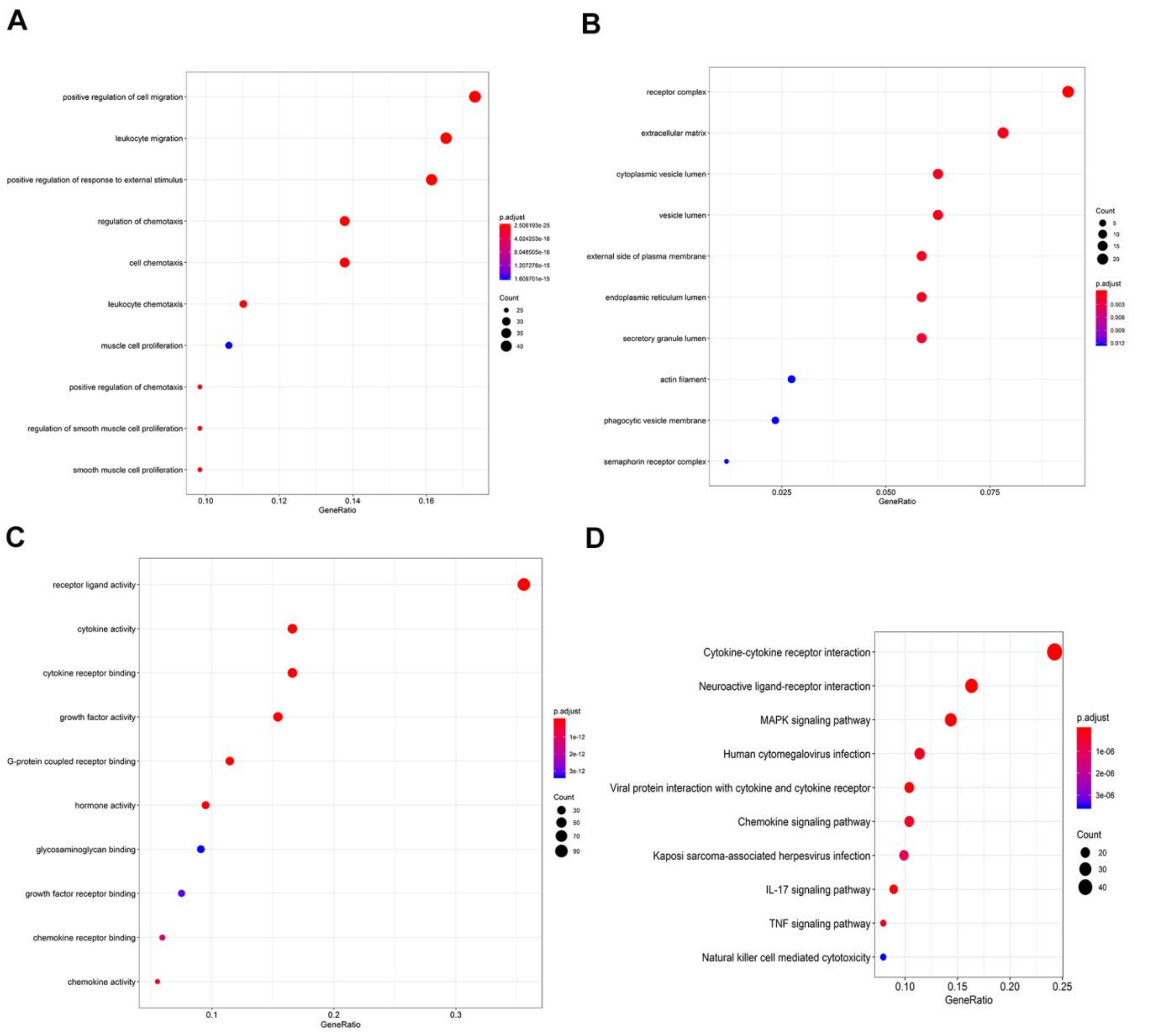

Supplementary Figure 2. Gene functional enrichment of differentially expressed genes. (**A**) The top ten most significant biological processes in the gene ontology. (**B**) The top ten most significant cellular components in the gene ontology. (**C**) The top ten most significant molecular functions in the gene ontology. (**D**) The top ten most significant KEGG pathways.

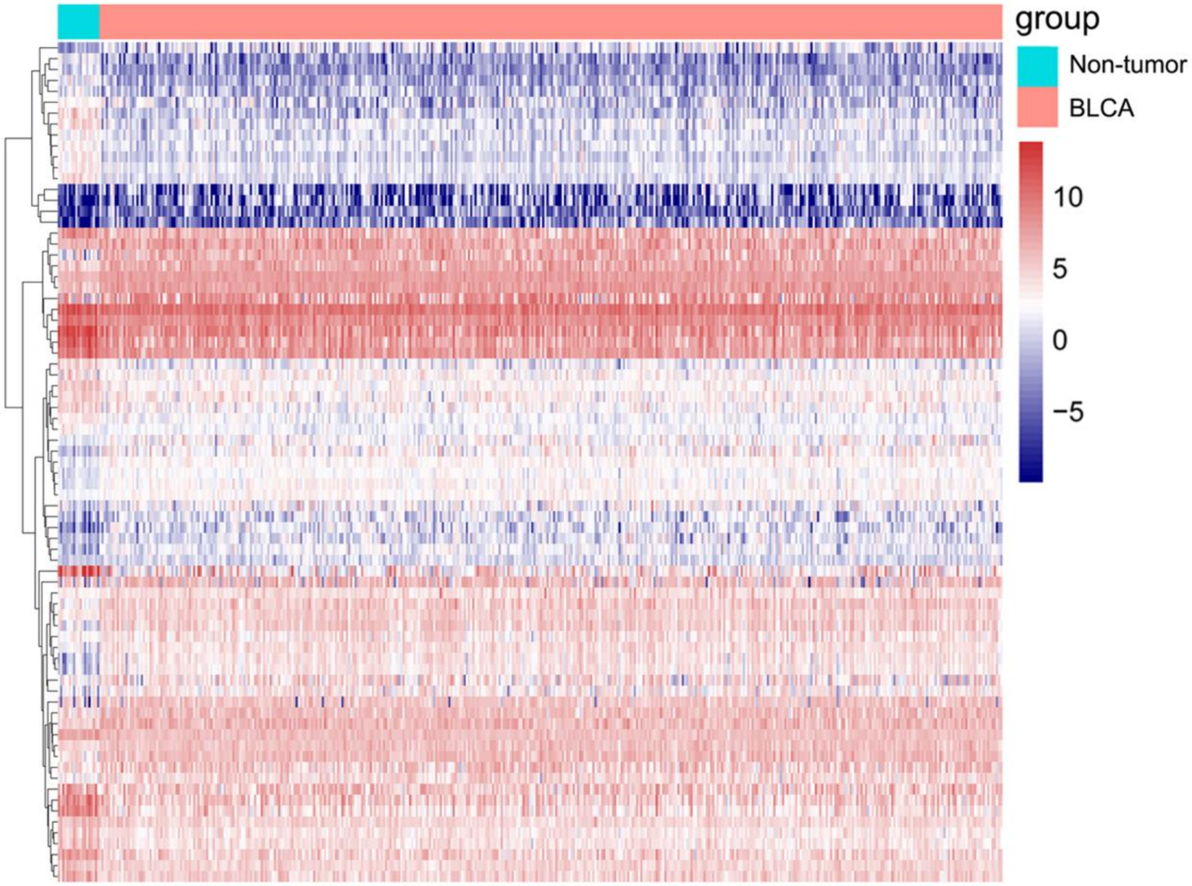

Supplementary Figure 3. Heatmap of differentially expressed TFs. Heatmap of differentially expressed TFs, the purple to red spectrum indicates low to high TF expression.
