# Supplementary material for: Identification of immune-related genes that predict prognosis and risk of bladder cancer: bioinformatics analysis of TCGA database
Source: Aging (Albany NY). 2021 Jul 30;13(15):19352–74. doi: 10.18632/aging.203333 (PMC8386543; doi:10.18632/aging.203333)
Supplement: Supplementary Tables [file aging-13-203333-s002.pdf]

SUPPLEMENTARY TABLES

Supplementary Table 1. Gene ontology analysis of differentially expressed immune-related genes.

GO	ID	Description	p.adjust	Count
Biological process	GO:0032103	positive regulation of response to external stimulus	2.51E-25	41
	GO:0050920	regulation of chemotaxis	1.58E-24	35
	GO:0060326	cell chemotaxis	3.91E-20	35
	GO:0030335	positive regulation of cell migration	7.70E-20	44
	GO:0050900	leukocyte migration	1.07E-18	42
	GO:0050921	positive regulation of chemotaxis	1.79E-18	25
	GO:0048660	regulation of smooth muscle cell proliferation	1.79E-17	25
	GO:0048659	smooth muscle cell proliferation	2.67E-17	25
	GO:0030595	leukocyte chemotaxis	4.67E-17	28
	GO:0033002	muscle cell proliferation	1.61E-15	27
Cellular component	GO:0043235	receptor complex	1.01E-07	24
	GO:0009897	external side of plasma membrane	8.15E-04	15
	GO:0031012	extracellular matrix	8.15E-04	20
	GO:0005788	endoplasmic reticulum lumen	8.15E-04	15
	GO:0060205	cytoplasmic vesicle lumen	8.15E-04	16
	GO:0031983	vesicle lumen	8.15E-04	16
	GO:0034774	secretory granule lumen	14.3E-03	15
	GO:0002116	semaphorin receptor complex	1.23E-02	3
	GO:0030670	phagocytic vesicle membrane	1.23E-02	6
	GO:0005884	actin filament	1.23E-02	7
Molecular function	GO:0048018	receptor ligand activity	1.51E-74	90
	GO:0008083	growth factor activity	2.53E-34	39
	GO:0005125	cytokine activity	6.50E-33	42
	GO:0005126	cytokine receptor binding	1.14E-28	42
	GO:0005179	hormone activity	7.88E-19	24
	GO:0001664	G-protein coupled receptor binding	2.52E-15	29
	GO:0008009	chemokine activity	3.33E-13	14
	GO:0042379	chemokine receptor binding	1.28E-12	15
	GO:0070851	growth factor receptor binding	3.03E-12	19
	GO:0005539	glycosaminoglycan binding	3.33E-12	23

Blue, green and red bars represent biological process, cellular component and molecular function, respectively.

Supplementary Table 2. Gene ontology analysis of prognosis-associated DEIRGs.

GO	ID	Description	p.adjust	Count
Biological process	GO:0048660	regulation of smooth muscle cell proliferation	3.64E-12	12
	GO:0048659	smooth muscle cell proliferation	3.64E-12	12
	GO:0033002	muscle cell proliferation	2.06E-10	12
	GO:0044706	multi-multicellular organism process	6.25E-09	11
	GO:0007565	female pregnancy	2.69E-08	10
	GO:0030335	positive regulation of cell migration	1.24E-07	13
	GO:0048661	positive regulation of smooth muscle cell proliferation	9.48E-07	7
	GO:0006874	cellular calcium ion homeostasis	3.24E-06	11
	GO:0055074	calcium ion homeostasis	3.76E-06	11
	GO:0002685	regulation of leukocyte migration	4.66E-06	8
Cellular component	GO:0009897	external side of plasma membrane	1.19E-02	6
	GO:0031012	extracellular matrix	1.40E-02	7
	GO:0031093	platelet alpha granule lumen	2.11E-02	3
	GO:0033116	Endoplasmic reticulum-Golgi intermediate compartment membrane	2.11E-02	3
	GO:0030670	phagocytic vesicle membrane	2.11E-02	3
	GO:0043034	costamere	2.12E-02	2
	GO:0031091	platelet alpha granule	2.50E-02	3
	GO:0060205	cytoplasmic vesicle lumen	2.50E-02	5
	GO:0031983	vesicle lumen	2.50E-02	5
	GO:0043235	receptor complex	3.16E-02	5
Molecular function	GO:0048018	receptor ligand activity	1.84E-11	16
	GO:0008083	growth factor activity	1.62E-06	8
	GO:0005125	cytokine activity	1.73E-04	7
	GO:0005126	cytokine receptor binding	6.01E-04	7
	GO:0005178	integrin binding	6.53E-04	5
	GO:0050431	transforming growth factor beta binding	6.53E-04	3
	GO:0005179	hormone activity	6.53E-04	5
	GO:0005539	glycosaminoglycan binding	7.57E-04	6
	GO:0019838	growth factor binding	8.01E-04	5
	GO:0008201	heparin binding	1.64E-03	5

Blue, green and red bars represent biological process, cellular component and molecular function, respectively.

Supplementary Table 3. Antibody information.

Antibody	Source	Catalog number	Dilution ratio
**ADIPOQ**	Abcam	ab133347	1:5000
**PPY**	Sigma	ZRB939	1:1000
**NAMPT**	Abcam	ab236874	1:1000
**TAP1**	Cell Signaling Technology	12341S	1:1000
**AHNAK**	Abcam	ab168104	1:1000
**OLR1**	Abcam	ab214427	1:1000
**PDGFRA**	Abcam	ab203491	1:1000
**IL34**	Abcam	ab101443	1:1000
**MMP9**	Abcam	ab76003	1:5000
**RAC3**	Abcam	ab129062	1:5000
**SH3BP2**	Absin	abs132638	1:1000
**GAPDH**	Abcam	ab9485	1:2500
